# Impact of pneumatic tube vs. bicycle courier transport on platelet aggregation: influence of sex and diabetes

**DOI:** 10.3389/fmed.2025.1738634

**Published:** 2026-01-30

**Authors:** Anna Hohneck, Elisabeth Kliemank, Simon Bach, Sebastian Brings, Lukas Seebauer, Mani Roshan, Zoltan Kender, Stefan Kopf, Norbert Frey, Markus Zorn, Stefan M. Woerner, Thomas Fleming, Julia Szendroedi

**Affiliations:** 1 Department of Endocrinology, Diabetology, Metabolism and Clinical Chemistry, Heidelberg University Hospital, Heidelberg, Germany; 2 German Center for Diabetes Research (DZD) Associated Partner Site Heidelberg, Heidelberg, Germany; 3 Department of Endocrinology and Diabetology, University Hospital Schleswig-Holstein, Luebeck, Germany; 4 Department of Cardiology, Angiology and Pneumology, University of Heidelberg, Heidelberg, Germany; 5 German Center for Cardiovascular Research (DZHK) Partner Site Heidelberg/Mannheim, Heidelberg, Germany

**Keywords:** bicycle courier, diabetes, platelet function, pneumatic tube system, sex-specific differences

## Abstract

**Aim:**

Pneumatic tube (PT) transport can impact platelet function, often altering platelet aggregation. Consequently, manual transport is frequently recommended to mitigate preanalytical effects on platelet function tests. However, comparative data between PT and bicycle courier (BC) transport remain limited, and it is unclear whether different agonist pathways or specific patient characteristics, such as sex and diabetes, modify transport-related effects.

**Methods:**

In this study, two S-Monovette® 3.2% citrate syringes of whole blood were collected from 96 participants (43 female participants, 53 male participants; median age: 63 years). Samples were transported simultaneously by PT or BC to the Central Laboratory of University Hospital Heidelberg. Platelet function was assessed through light transmission aggregometry (LTA) using five diagnostically established agonists, including ADP, arachidonic acid, ristocetin, collagen, and epinephrine.

**Results:**

BC transport was associated with small but statistically significant reductions in platelet aggregation compared with PT for several agonists: ADP (−2.5%, *p* = 0.02), arachidonic acid (−1.0%, *p* = 0.006), ristocetin (−2.0%, *p* = 0.003), and collagen (−3.0%, *p* = 0.002), while epinephrine-induced aggregation was unaffected (*p* = 0.58). These reductions were more pronounced in female patients and individuals with diabetes, particularly for collagen-induced aggregation (up to −3.5%, *p* = 0.02). Epinephrine-induced aggregation was unaffected by transport mode (*p* = 0.58). Females patients showed higher aggregation responses overall, especially to arachidonic acid (*p* = 0.02 vs. males). Among participants, antiplatelet drugs markedly inhibited ADP, arachidonic acid, and epinephrine-induced aggregation but did not meaningfully affect collagen or ristocetin responses.

**Conclusion:**

PT transport resulted in minor increases in platelet aggregation compared with BC transport, which are unlikely to be clinically relevant for the majority of patients but may influence interpretation in selected subgroups, particularly in individuals with heightened platelet reactivity or borderline test results. The observed transport-related differences in female patients and individuals with diabetes suggest increased platelet mechanosensitivity in these groups and warrant confirmation in larger, dedicated studies.

## Introduction

Platelets play a key role in hemostasis and are central to the regulation of bleeding and thrombosis processes (1). Measuring platelet function is therefore crucial for assessing bleeding risk, particularly before surgical procedures or in cases of suspected hemorrhagic disorders (2). Platelet function tests are used to detect congenital or acquired platelet dysfunctions and to monitor the effectiveness of antiplatelet therapy. However, preanalytical factors—including the method of blood sample transport—can significantly affect test outcomes and may lead to misclassification of platelet function if not carefully controlled (3).

Pneumatic tube (PT) systems are commonly used in hospitals for rapid and automated transport of blood samples. Several studies have reported that PT transport can alter platelet function or global hemostasis assays, presumably through shear forces, vibration, and repeated acceleration–deceleration during transit, which may activate or partially exhaust platelets before analysis (4–6). In parallel, patients with diabetes mellitus exhibit enhanced platelet reactivity and baseline activation, driven by chronic hyperglycemia, insulin resistance, oxidative stress, and glycation of platelet surface proteins, which contributes to their elevated atherothrombotic risk (7, 8). Female individuals also tend to show higher platelet reactivity than men, including greater fibrinogen binding, higher surface receptor expression, and stronger responses to certain agonists, which may partly underlie sex-specific differences in cardiovascular risk (9–11). These abnormalities imply that platelets in diabetes and in females may be particularly vulnerable to additional mechanical stimuli, such as those imposed by sample transport, and support our focus on transport-related mechanosensitivity in these high-risk groups.

Consequently, the International Council for Standardization in Hematology (ICSH) recommends validating PT systems before use for platelet function testing to mitigate preanalytical effects (12). Manual transport is often preferred to preserve sample integrity, but it is rarely feasible in larger hospital settings with centralized laboratories. Other transport methods, such as bicycle courier (BC), may represent practical alternatives; however, systematic, paired comparisons of PT with alternative transport modes such as BC and their potential interaction with sex and diabetes remain scarce.

The primary aim of this study was to assess whether PT transport is non-inferior to BC transport with respect to platelet aggregation measured by LTA in a clinical cohort. In addition, pre-specified exploratory analyses by sex and diabetes status were conducted to generate hypotheses about potential modifiers of transport-related effects on platelet function.

## Methods

### Participants and study design

#### Study design and ethical approval

This was a single-center, observational cross-sectional study embedded in the “Heidelberg Study on Diabetes and Complications” (Heist-DiC) conducted at the Department of Internal Medicine I, University Hospital Heidelberg, between 1 December 2022 and 30 March 2023. The protocol was approved by the local ethics committee (Medical Ethics Commission I, Heidelberg University, Approval No. S-383/2016), and all participants provided written informed consent in accordance with the Declaration of Helsinki and applicable data protection regulations.

An *a priori* sample size calculation was performed for a non-inferiority comparison of platelet aggregation between PT and BC transport, assuming a non-inferiority margin of −5%, an expected difference of −2.5%, a one-sided alpha of 0.025, and 90% power, resulting in a required sample size of 98 participants; to account for potential dropouts or technical issues, 100 participants were recruited.

#### Inclusion and exclusion criteria

All adults aged 18–85 years participating in Heist-DiC were eligible for this sub-study. Exclusion criteria at inclusion or at the respective visit were: secondary diabetes mellitus [American Diabetes Association (ADA) criteria]; HbA1c ≥ 9.5% at inclusion; current pregnancy; acute infection or fever; immunosuppressive therapy; severe psychiatric illness requiring treatment; dependence on alcohol or other drugs; severe heart, kidney, or liver disease; malignant cancer in the last 5 years; infectious diseases (hepatitis B, C, and E and HIV); autoimmune diseases requiring immunosuppressive therapy; current participation in an intervention study; anemia; or bone marrow disorders.

#### Grouping of patients

For the present analysis, participants were grouped according to sex (female vs. male) and diabetes status (diabetes mellitus vs. no diabetes mellitus) based on established clinical criteria and medical history. These groupings were used for pre-specified exploratory subgroup analyses to investigate potential modifiers of transport-related effects on platelet aggregation. Additional descriptive groupings (e.g., antiplatelet therapy, hypertension, and statin use) are reported in the baseline characteristics but were not used for formal interaction testing.

#### Sample handling and storage

Venous blood was drawn under fasting conditions into two S-Monovette® 3.2% citrate tubes per participant by experienced staff following European Federation of Clinical Chemistry and Laboratory Medicine (EFLM) recommendations and stored at room temperature for up to 120 min before transport. The samples were then transported either via the hospital’s central PT system or by BC over routes of similar length (approximately 500–600 m) and speed (PT about 4 m/s ≈ 14.4 km/h; BC about 15 km/h). For PT transport, tubes were loosely wrapped in a plastic bag, placed in cushioned carriers, and sent through a route with multiple vertical and horizontal segments and bends without active temperature control. For BC transport, the tubes were collected at the same time as PT dispatch and carried upright in a rack inside an insulated Styrofoam box without active cooling to protect them from direct sunlight and mechanical shocks and to minimize differences in time from blood draw to analysis.

### Platelet function testing

#### Preparation of platelet-rich and platelet-poor plasma

Platelet-rich plasma (PRP) was prepared by centrifuging citrated whole blood at 150 g for 10 min at room temperature. Platelet-poor plasma (PPP) was obtained by further centrifugation of the remaining supernatant at 1,500 *g* for 10 min. The optical density of PPP was set as 100% aggregation, and PRP served as the 0% baseline. Platelet concentration in PRP had to be ≥75/nl for measurements to be considered valid, in line with current recommendations for light transmission aggregometry (LTA) (13); this threshold was checked using routine hematology analyzers and is consistent with previously published standardization efforts.

#### Light transmission aggregometry procedure

Platelet function was assessed by LTA using a PAP-8 aggregometer (Moelab, Germany), following Scientific and Standardization Committee/International Society on Thrombosis and Haemostasis (SSC/ISTH) and German Society for Thrombosis and Haemostasis Research (GTH) standardization guidance. PRP samples were equilibrated at 37 °C before measurement, and aggregation was recorded under continuous stirring in individual cuvettes. The instrument was pre-warmed according to the manufacturer’s instructions before each measurement run. Agonists were added separately to aliquots of PRP, and aggregation was monitored for 10 min after stimulation; the maximum change in light transmission during this period was recorded as the aggregation response. The samples were analyzed in series but not pooled; all measurements for a given participant (PT and BC) were performed within the same analytical session to minimize inter-run variability.

#### Agonists and choice of stimulants

In accordance with SSC/ISTH recommendations, five agonists were used at standard final concentrations: adenosine-5′-diphosphate (ADP, 20 μM), arachidonic acid (1.64 mM), ristocetin (1.5 mg/mL), collagen (0.19 mg/mL), and epinephrine (100 μM). These agonists cover distinct signaling pathways relevant to primary hemostasis, ADP-dependent signaling, thromboxane A2 synthesis, von Willebrand factor-mediated platelet adhesion, collagen receptor activation, and epinephrine-induced aggregation. Thrombin was not used as an agonist because high-dose thrombin induces near-maximal, non-physiological platelet activation *in vitro* and is less informative for detecting subtle transport-related differences or pharmacological inhibition in standard diagnostic LTA protocols.

A negative control sample from a healthy individual without antiplatelet therapy or von Willebrand disease was measured once weekly to monitor assay performance; intra-laboratory coefficients of variation for the five agonists were approximately 6–9% for ADP, epinephrine, arachidonic acid, and collagen, and they were approximately 20% for ristocetin, which is consistent with published inter-laboratory quality assessments.

#### Statistical analysis

Data are presented as mean ± standard deviation, median with interquartile range (IQR), or frequency (percentage), as appropriate. For continuous variables that deviate from a normal distribution, the IQR is defined as the distance between the 25th and 75th percentiles and reflects the dispersion of the central 50% of the data. The D’Agostino–Pearson omnibus normality test was performed for distribution testing. Continuous variables were compared using a two-tailed paired Student’s *t*-test for parametric and a Wilcoxon signed-rank test for non-parametric variables. Comparisons between independent groups were carried out with the Mann–Whitney U-test. Bland–Altman analyses were performed to assess the agreement between platelet aggregation measurements obtained by LTA for different agonists, comparing PT and BC transport methods. The degree of agreement was quantified using the concordance correlation coefficient (CCC). Pre-specified exploratory subgroup analyses by sex (female vs. male) and diabetes status (diabetes vs. no diabetes) were conducted to generate hypotheses about potential modifiers of transport-related effects on platelet aggregation, but the study was not powered for subgroup-specific effects or interaction testing. Consequently, these subgroup results are underpowered for detecting or excluding small to moderate interaction effects and should be regarded as hypothesis-generating, with associated *p*-values and effect estimates interpreted cautiously. Statistical analyses were performed using the Statistical Package for Social Sciences (SPSS version 29.0, Chicago, Illinois, USA) and GraphPad Prism 9.5 (GraphPad Software, Inc., California, USA). Two-sided *p*-values <0.05 were considered statistically significant.

## Results

### Baseline characteristics

Platelet aggregation data were obtained from 96 participants (43 male and 53 female) with a median age of 63 years [IQR: 52–72]. Female participants were significantly older than male participants [median 67 years (54; 76) vs. median 59 years (50; 67), *p* = 0.02], and all were postmenopausal. The cohort had a median body mass index (BMI) of 27 kg/m^2^ [IQR 24–32]. Diabetes mellitus was present in 48 participants (50.0%), with 30 (31.3%) receiving oral antidiabetic therapy, 26 (27.1%) receiving insulin, and nine (9.4%) receiving glucagon-like peptide 1 (GLP-1) receptor agonists. Age and BMI did not differ significantly between individuals with and without diabetes [age of individuals without diabetes median 60 years (52; 69) vs. individuals with diabetes median 65 years (53; 73), *p* = 0.80; BMI of individuals without diabetes 27 kg/m^2^ (24; 32) vs. individuals with diabetes 27 kg/m^2^ (25; 31), *p* = 0.77]. Hypertension was diagnosed in 51 individuals (53.1%). Antiplatelet drugs were taken by 27 individuals (28.1%), more frequently in females than in males [20 (37.7%) vs. 7 (16.3%), *p* = 0.02]. A total of 26 individuals received acetylsalicylic acid (ASA) monotherapy, and one individual received dual antiplatelet therapy (DAPT) with ASA and clopidogrel. Statins were taken by 34 individuals (35.4%), and 13 individuals (13.5%) reported using non-steroidal anti-inflammatory drugs (NSAIDs) within the last 7 days. The median platelet count was within the normal range at 231/nl [IQR 200–278], with female patients showing a significantly lower platelet count, which is physiological [median 220/nl (176; 251) vs. median 248/nl (212; 302), *p* = 0.02]. No coagulation disorders were observed, with a median Quick of 104% [IQR 98–114] and an international normalized ratio (INR) of 1.0 [IQR: 0.9–1.0] (Table 1).

**Table 1:** Baseline characteristics of the study population.

	*N* = 96	Male participants (*n* = 43, 44.8%)	Female participants (*n* = 53, 55.2%)	*p*-value
Anthropometric data				
Age, years	63 (52; 72)	59 (50; 67)	67 (54; 76)	0.02
BMI, kg/m^2^	27 (24; 32)	27 (24; 34)	27 (25; 30)	0.22
Comorbidities				
Diabetes mellitus, %	48 (50.0)	20 (46.5)	28 (52.8)	0.54
Hypertension, %	51 (53.1)	21 (48.8)	30 (56.6)	0.45
Medication				
OAD, %	30 (31.3)	11 (25.6)	19 (35.8)	0.29
Insulin, %	26 (27.1)	11 (25.6)	14 (26.4)	0.93
GLP-1-RA, %	9 (9.4)	4 (9.3)	5 (9.4)	0.98
Antiplatelet drugs, %	27 (28.1)	7 (16.3)	20 (37.7)	0.02
Statins	34 (35.4)	13 (30.2)	21 (39.6)	0.28
NSAID	13 (13.5)	8 (18.6)	5 (9.4)	0.20
ASA	26 (27.1)	7 (16.3)	19 (35.8)	0.03
DAPT (ASA + clopidogrel)	1 (1.0)	0 (0.0)	1 (1.9)	0.37
Laboratory values				
Platelet count, /nl	231 (200; 278)	248 (212; 302)	220 (176; 251)	0.02
Quick, %	104 (98; 114)	109 (100; 117)	103 (94; 111)	0.42
INR	1.0 (0.9; 1.0)	1.0 (0.9; 1.0)	1.0 (1.0; 1.0)	0.43

Baseline demographic and clinical data of all participants (*N* = 96). Data are presented as median (IQR) or numbers (percentages). ASA, acetylsalicylic acid; BMI, body mass index; DAPT, dual antiplatelet therapy; GLP-1-RA, glucagon-like peptide-1 receptor agonist; NSAID, non-steroidal anti-inflammatory drug; OAD, oral antidiabetic drugs.

### Effect of transport method on platelet aggregation

**Figure 1:**
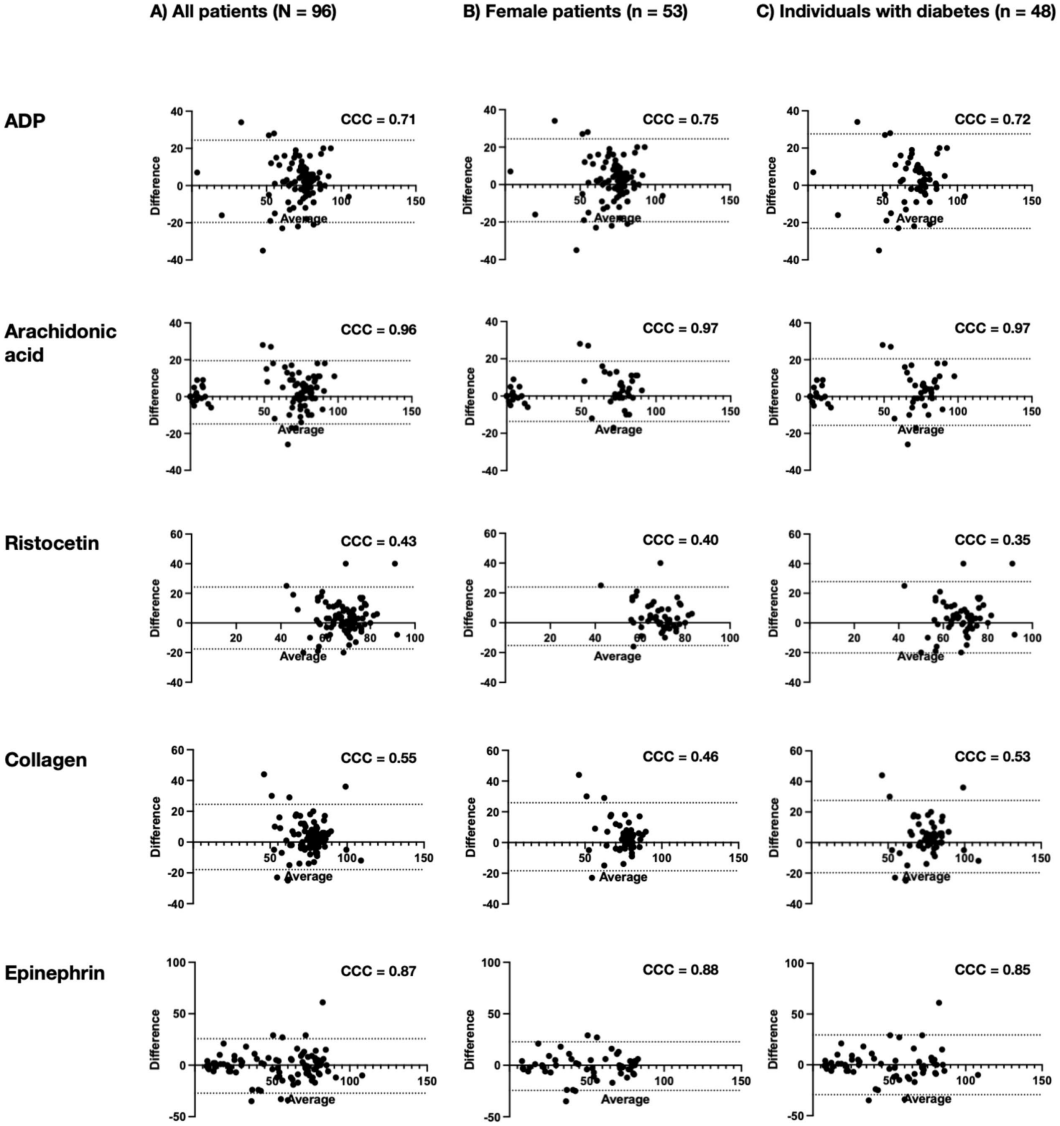
Bland–Altman plot. Bland–Altman plots comparing the impact of two transport methods for platelet aggregation responses to five agonists (ADP, arachidonic acid, ristocetin, collagen, and epinephrine) across three subgroups: **(A)** All patients (*N* = 96), **(B)** female patients (*n* = 53), and **(C)** individuals with diabetes (*n* = 48). Each plot shows the mean of both transport methods on the *x*-axis and their differences on the *y*-axis. Dashed lines indicate the mean difference and 95% limits of agreement. Concordance correlation coefficients (CCC) quantify the level of agreement for each agonist and subgroup.

Compared to PT transport, BC transport was associated with small but statistically significant reductions in platelet aggregation of several agonists (Table 2; Figure 1A). For ADP, aggregation levels were 76% [IQR: 68–80] following PT transport versus 74% [IQR: 66–79] with BC transport (*p* = 0.02), corresponding to a median reduction of 2.5%. A similar pattern was observed for arachidonic acid, with aggregation levels of 72% [IQR: 11–80] after PT transport and 72% [IQR: 8–79] after BC transport, reflecting a 1.0% reduction (*p* = 0.006). Ristocetin-induced aggregation decreased from 69% [IQR 66–74] with PT to 68% [IQR 62–72] with BC (*p* = 0.003), while collagen-induced aggregation declined from 78% [IQR: 71–83] to 76% [IQR 68–81] (*p* = 0.002), representing a 2.0–3.0% reduction depending on the agonist. In contrast, epinephrine-induced aggregation was not significantly affected by transport method, with values of 57% [IQR: 30–75] for PT transport and 60% [IQR 30–76] for BC transport (*p* = 0.58).

**Table 2:** Comparison of agonist-induced platelet aggregation according to the transport method.

Agonist	PT, aggregation (%)	BC, aggregation (%)	Percentage of reduction (%), PT vs. BC	*p*-value
All patients (*N* = 96)				
ADP	76 (68; 80)	74 (66; 79)	−2.5 (−8.3; 3.0)	0.02
Arachidonic acid	72 (11; 80)	72 (8; 79)	−1.0 (−7.0; 1.0)	0.006
Ristocetin	69 (66; 74)	68 (62; 72)	−2.0 (−9.0; 2.0)	0.003
Collagen	78 (71; 83)	76 (68; 81)	−3.0 (−7.0; 3.0)	0.002
Epinephrine	57 (30; 75)	60 (30; 76)	0.0 (−5.0; 7.3)	0.58

Comparison of platelet aggregation (%) and percentage reduction for pneumatic transport (PT) and bicycle courier (BC) transport in the total cohort (*N* = 96). Aggregation responses to ADP, arachidonic acid, ristocetin, and collagen were slightly but significantly reduced following BC transport, while epinephrine-induced aggregation remained unaffected. Data are presented as median (IQR). ADP, adenosine-5′-diphosphate.

### Sex-related differences in platelet aggregation

Sex-related differences in platelet function were observed independently of the transport method (Table 3; Figure 1B). For arachidonic acid, females participants showed consistently higher aggregation values than male participants in both transport conditions: under PT transport, female values were 74% [IQR: 64–82] compared to 68% [IQR: 5–79] in males (*p* = 0.02); under BC transport, the difference persisted with female values at 75% [IQR: 62–80] versus 63% [IQR: 5–78] in males (*p* = 0.02). A similar trend was seen for epinephrine-induced aggregation, with higher values in female participants for both PT [69% (IQR: 41–81)] and BC [71% (IQR: 48–77)] transport compared to male participants [PT: 49% (IQR 29–72); BC: 54% (IQR 27–74)], although the differences did not reach statistical significance (*p* = 0.06 for both) and were attenuated after adjustment for age. No significant sex-related differences were observed for the remaining agonists, including ADP, ristocetin, and collagen. Furthermore, female participants in our cohort were older than male participants, and age differences may partly account for the observed sex-related patterns in platelet aggregation, even after adjustment in exploratory analyses. Residual confounding by age and other unmeasured factors cannot be excluded.

**Table 3:** Sex-specific comparison of agonist-induced platelet aggregation.

Agonist	Male participants (*n* = 43, 44.8%)	Female participants (*n* = 53, 55.2%)	*p*-value	*p*-value adjusted for age
PT				
ADP	76 (71; 81)	76 (65; 80)	0.07	0.63
Arachidonic acid	74 (64; 82)	68 (5; 79)	0.02	0.04
Ristocetin	69 (63; 74)	69 (66; 74)	0.93	0.07
Collagen	78 (71; 85)	78 (75; 82)	0.26	0.052
Epinephrine	69 (41; 81)	49 (29; 72)	0.06	0.96
BC				
ADP	74 (70; 80)	72 (62; 78)	0.06	0.07
Arachidonic acid	75 (62; 80)	63 (5; 78)	0.02	0.03
Ristocetin	68 (63; 72)	68 (59; 73)	0.11	0.052
Collagen	76 (68; 81)	76 (69; 80)	0.17	0.10
Epinephrine	71 (48; 77)	54 (27; 74)	0.06	0.93

Comparison of pneumatic transport (PT) and bicycle courier (BC) platelet aggregation responses between male (*n* = 43) and female participants (*n* = 53). Females showed higher arachidonic acid-induced aggregation in both transport conditions, whereas other agonists exhibited no significant sex-related differences after adjustment for age. Data are presented as median (IQR). ADP, adenosine-5′-diphosphate.

### Differences according to diabetes status

Differences in platelet aggregation were also observed between individuals with diabetes and glucose-tolerant participants (Table 4; Figure 1C). For epinephrine-induced aggregation, individuals with diabetes exhibited significantly lower values than those without diabetes, regardless of transport method. Under PT transport, aggregation was 45% [IQR 21–70] in participants with diabetes compared to 69% [IQR: 47–79] in glucose-tolerant individuals (*p* = 0.002); under BC transport, values were similarly reduced [52% (IQR 23–73) vs. 71% (IQR 50–79), *p* = 0.01]. A comparable pattern was observed for ADP-induced aggregation in the BC group, where diabetic individuals showed lower values [71% (IQR 62–78)] than those without diabetes [76% (IQR 68–79), *p* = 0.04], although the difference was not statistically significant under PT conditions (*p* = 0.07). Aggregation responses to ristocetin and collagen were also modestly lower in individuals with diabetes, with collagen-induced aggregation decreasing from 78% [IQR 72–84] to 75% [IQR 69–81] (*p* = 0.02) and ristocetin-induced aggregation from 69% [IQR 65–74] to 67% [IQR 60–72] (*p* = 0.03) following BC transport. These findings suggest that diabetes is associated with attenuated platelet responsiveness to certain agonists, particularly those involved in shear-sensitive or generalized activation pathways.

**Table 4:** Platelet aggregation in individuals with diabetes.

Agonist	PT, aggregation (%)	BC,aggregation(%)	Percentage of reduction (%), PT vs. BC	*p*-value
Individuals with diabetes (*n* = 48)				
ADP	76 (67; 79)	72 (62; 78)	−2.5 (−9.0; 4.3)	0.19
Arachidonic acid	68 (5; 80)	66 (4; 78)	0.0 (−7.0; 1.0)	0.047
Ristocetin	69 (65; 74)	67 (60; 72)	−2.5 (−9.3; 2.0)	0.03
Collagen	78 (72; 84)	75 (69; 81)	−3.5 (−7.0; 2.0)	0.02
Epinephrine	48 (23; 71)	53 (24; 73)	0.0 (−5.0; 7.3)	0.99

Agonist-induced platelet aggregation (%) and percentage reduction between pneumatic transport (PT) and bicycle courier (BC) conditions among individuals with diabetes (*n* = 48). Aggregation responses to collagen, ristocetin, and arachidonic acid were modestly reduced following BC transport. Data are presented as median (IQR). ADP, adenosine-5′-diphosphate; ASA, acetylsalicylic acid; DAPT, dual antiplatelet therapy.

### Agreement between transport methods and effect of antiplatelet therapy

A high degree of agreement between PT and BC transport was observed for epinephrine- and arachidonic acid-induced aggregation, with concordance correlation coefficients (CCC) of ≥0.85 and ≥0.96, respectively (Figures 1A–C). This indicates that these pathways are comparatively less susceptible to transport-induced mechanical stress. Aggregation responses to ADP and epinephrine were significantly inhibited by antiplatelet medication, while responses to collagen and ristocetin were largely unaffected (Supplementary Table 1). Among individuals receiving antiplatelet therapy, no significant differences in platelet aggregation were observed between transport methods, further supporting the overall robustness of the findings in clinically relevant subgroups.

## Discussion

This study investigated the impact of two hospital-based transport methods—pneumatic tube (PT) and bicycle courier (BC)—on platelet aggregation results and found that both modes are generally suitable for sample delivery to centralized laboratories. Although BC transport resulted in small but statistically significant reductions in aggregation for several agonists, including ADP, arachidonic acid, ristocetin, and collagen, the magnitude of these differences was modest (1.0–3.0%) and unlikely to be clinically relevant in the majority of cases. However, subgroup analyses revealed more pronounced effects in females and individuals with diabetes, suggesting that certain patient characteristics may increase susceptibility to transport-induced mechanical influences on platelet function.

The observed reductions with BC transport may reflect subtle differences in the mechanical stress profile experienced by blood samples during transit. PT systems, while rapid and widely adopted, involve high acceleration and deceleration forces, vibration, and abrupt directional changes, which can stress platelets and potentially alter their activation state. Prior studies have shown that PT systems can impair platelet aggregation, either by triggering shear-induced activation or by inducing platelet exhaustion and receptor desensitization (4–6). In contrast, BC transport may offer a gentler mechanical environment, although it introduces variability through environmental factors such as temperature and handling conditions. The higher concordance observed for arachidonic acid and epinephrine-induced aggregation (concordance correlation coefficients ≥0.96 and ≥0.85, respectively) suggests that these pathways are relatively robust to such transport effects. By contrast, aggregation responses to ADP, ristocetin, and collagen—pathways more sensitive to shear stress or surface receptor engagement—showed greater variability, consistent with mechanosensitive behavior (14, 15).

Subgroup analyses highlighted important patient-specific modifiers of transport sensitivity. Female participants exhibited consistently higher platelet aggregation values, particularly in response to arachidonic acid and epinephrine, regardless of transport mode. These findings align with prior research demonstrating increased platelet reactivity in female individuals, including greater fibrinogen binding, higher surface receptor expression, and enhanced response to certain agonists (10, 11). Although all female participants in this study were postmenopausal, residual sex-related differences may persist and influence platelet activation dynamics. The more pronounced reductions in collagen and ristocetin aggregation in females with BC transport may reflect increased shear sensitivity or alterations in mechanosensitive signaling via glycoprotein VI or integrin α2β1 (16).

Similarly, individuals with diabetes showed altered aggregation responses, with particularly reduced reactivity to epinephrine across both transport methods. This observation is consistent with prior studies describing platelet dysfunction in diabetes, including impaired calcium signaling, altered membrane fluidity, and enhanced oxidative stress (7, 9). Chronic hyperglycemia has also been linked to abnormal platelet activation profiles, which may predispose diabetic platelets to altered responsiveness under mechanical strain. The transport-related reductions in collagen and ristocetin responses in this subgroup may therefore reflect a greater vulnerability to preanalytical variation, possibly due to pre-activated or dysfunctional platelet populations.

The subgroup findings regarding sex and diabetes should be interpreted with caution, as the study was not specifically powered to detect interaction effects, and multiple comparisons increase the risk of chance findings. Accordingly, these results are best viewed as preliminary, hypothesis-generating signals that warrant confirmation in larger, dedicated studies with adequate power and prespecified interaction testing.

The role of antiplatelet therapy was also considered. As expected, aggregation responses to ADP and arachidonic acid were significantly suppressed in participants taking antiplatelet drugs, confirming assay sensitivity to P2Y12 inhibition and COX blockade (17). In contrast, collagen- and ristocetin-induced aggregation remained largely unaffected by antiplatelet medication, consistent with previous observations that these pathways reflect more general aspects of primary hemostasis (14). Importantly, among participants receiving antiplatelet drugs, no significant transport-related differences were observed, supporting the robustness of both PT and BC transport in clinical contexts involving pharmacological platelet inhibition.

These findings have practical implications for laboratory operations in large clinical settings. While PT systems offer speed and automation, their mechanical forces can variably affect sample integrity (18, 19). Institutional differences in PT system design, tube speed, and landing dynamics further complicate reproducibility, reinforcing current recommendations by the ICSH to validate platelet function under local conditions before adopting PT transport (12). In contrast, BC transport—though more labor-intensive—may be suitable for long-distance in-hospital transport with reduced mechanical stress. Nevertheless, external conditions such as ambient temperature and humidity during BC transit must be considered, as they can also impact platelet function (20, 21).

Overall, this study underscores the importance of preanalytical conditions in the interpretation of platelet function tests. While the observed differences between transport methods were modest, they may become clinically relevant in selected patient populations, particularly those with enhanced platelet sensitivity, such as postmenopausal women or individuals with diabetes. Future research may focus on refining transport protocols, exploring platelet-preserving containers, or validating point-of-care alternatives for time-sensitive diagnostics (22–24). Ultimately, a deeper understanding of how mechanical transport interacts with platelet biology could help minimize diagnostic variability and improve patient care in hematologic and cardiovascular risk assessment.

### Strengths and limitations

This study has several strengths and limitations. The main strengths are the rigorous experimental design and the direct clinical relevance of the research question. The paired-sample, simultaneous-transport protocol effectively controls for inter-individual variability and provides a robust internal control for comparing PT and BC transport, while the use of five platelet agonists targeting distinct signaling pathways enables a comprehensive assessment of platelet function beyond single-agonist metrics.

However, the study was prospectively powered for the overall non-inferiority comparison between PT and BC, but not for subgroup analyses by sex or diabetes status, which therefore have limited statistical power and an increased risk of both false positive and false negative findings. In addition, the exploratory subgroup analyses involved multiple comparisons and potential confounding by age and other clinical factors, and formal interaction tests were not powered to reliably distinguish true effect modification from random variation, so the observed subgroup patterns should be regarded as preliminary and hypothesis-generating and require confirmation in larger, dedicated studies.

## Conclusion

BC transport resulted in statistically significant differences in aggregation for several agonists, although the absolute effect sizes were modest, typically in the range of 1–3 percentage points, and thus comparable to the analytical and biological variability reported for LTA in method comparison and transport studies. On this basis, these differences are unlikely to be clinically relevant for the majority of patients but could influence interpretation in borderline cases or in individuals with particularly heightened or attenuated platelet reactivity. Given the importance of accurate platelet function testing in clinical decision-making, especially in patients receiving antiplatelet therapy, the choice of transport method warrants consideration. While BC transport may offer slightly greater stability for mechanosensitive pathways, both methods are generally reliable when preanalytical variables are well-controlled. Ultimately, transport method is only one of several factors—alongside sample handling, temperature, time to analysis, and patient characteristics such as sex—that contribute to variability in platelet function results. Further research is needed to assess whether these transport-related differences translate into clinically meaningful outcomes and to develop best practice guidelines for samples in specialized coagulation diagnostics.

## Data Availability

The raw data supporting the conclusions of this article will be made available by the authors, without undue reservation.
