# Supplementary material for: Impact of pneumatic tube vs. bicycle courier transport on platelet aggregation: influence of sex and diabetes
Source: Front Med (Lausanne). 2026 Jan 30;12:1738634. doi: 10.3389/fmed.2025.1738634 (PMC12902833; doi:10.3389/fmed.2025.1738634)
Supplement: Supplementary file 1 [file Table_1.docx]

**Supplementary Table 1. Antiplatelet drugs**

|  | **Patients with antiplatelet drugs (n = 27)** | **Patients without antiplatelet drugs (n = 69)** | **p-value** Mann-Whitney-U-test |
| --- | --- | --- | --- |
| **ADP** | | | |
| PT | 74 (61; 80) | 78 (70; 80) | 0.004 |
| BC | 70 (64; 77) | 74 (68; 80) | 0.03 |
| p-value | 0.84 | 0.02 |  |
| **Arachidonic acid** | | | |
| PT | 4 (0; 10) | 76 (69; 82) | < 0.0001 |
| BC | 3 (0; 6) | 75 (68; 80) | < 0.0001 |
| p-value | 0.78 | 0.01 |  |
| **Ristocetin** | | | |
| PT | 69 (66; 74) | 70 (66; 74) | 0.83 |
| BC | 66 (63; 72) | 68 (61; 73) | 0.80 |
| p-value | 0.11 | 0.02 |  |
| **Collagen** | | | |
| PT | 77 (68; 81) | 78 (73; 84) | 0.25 |
| BC | 74 (71; 78) | 77 (68; 82) | 0.49 |
| p-value | 0.35 | 0.002 |  |
| **Epinephrine** | | | |
| PT | 23 (18; 45) | 70 (49; 77) | < 0.0001 |
| BC | 26 (16; 48) | 71 (55; 77) | < 0.0001 |
| p-value | 0.53 | 0.28 |  |

Comparison of platelet aggregation responses between participants receiving antiplatelet medication (n = 27) and those without therapy (n = 69). Individuals on ASA or DAPT showed markedly reduced aggregation in response to ADP and epinephrine, whereas collagen‑ and ristocetin‑induced aggregation remained largely unaffected. Data are presented as median (IQR). Comparisons between PT and BC conditions were conducted using the Wilcoxon signed‑rank test; intergroup comparisons with or without antiplatelet therapy were performed using the Mann‑Whitney‑U‑test. ADP, adenosine‑5’‑diphosphate.
